# Prone Position Video Laryngoscopy for the Successful Intubation of Penetrating Back Injury: A Report of Two Cases

**DOI:** 10.7759/cureus.76119

**Published:** 2024-12-21

**Authors:** Nisha Jain, Aayush Kulshrestha, Navdeep Kaur, Abhishek Singh

**Affiliations:** 1 Anesthesiology, All India Institute of Medical Sciences, New Delhi, IND

**Keywords:** airway management, difficult airway management, intubation, prone position surgery, video laryngoscopy

## Abstract

Airway management in the prone position presents significant challenges and carries the risk of encountering a difficult airway situation. Here, we present two adults who sustained traumatic knife injuries to the back and required surgical intervention. Due to the potential life-threatening complications associated with dislodging the knife, traditional supine and lateral decubitus positions were not feasible for airway management. To overcome this obstacle, we employed a video laryngoscope for intubation in the prone position. We discuss the challenges of airway management in such cases, the decision-making process behind choosing the prone position, and the outcomes of this novel approach in facilitating successful intubation.

## Introduction

Penetrating trauma to the back, especially when involving critical structures such as the thorax, neck, or airway, presents significant challenges in the acute setting. Airway management in trauma patients positioned prone poses particular difficulties, including challenges with bag-mask ventilation, inability to insert a laryngeal mask airway (LMA), and difficult endotracheal intubation. Video laryngoscopy (VL) has significantly impacted airway management, with multiple studies confirming its effectiveness in managing difficult airways clinically [1]. However, its application in the prone position, especially in cases of penetrating back trauma, remains underreported.

Here, we present the anesthetic management of two adult patients with traumatic knife injuries to the upper dorsal surface of the body, which required surgical intervention. Due to the potential for additional damage from dislodging the knife, the supine and lateral positions were deemed unsuitable for airway management. Given the difficulties associated with airway management in the prone position, we employed VL under general anesthesia to secure the airway. Prior written consent was obtained from both patients for the publication of this case report. The following case highlights the use of VL in the prone position to successfully manage the airway of patients with knife injuries to the back.

## Case presentation

Case 1

**Figure 1:**
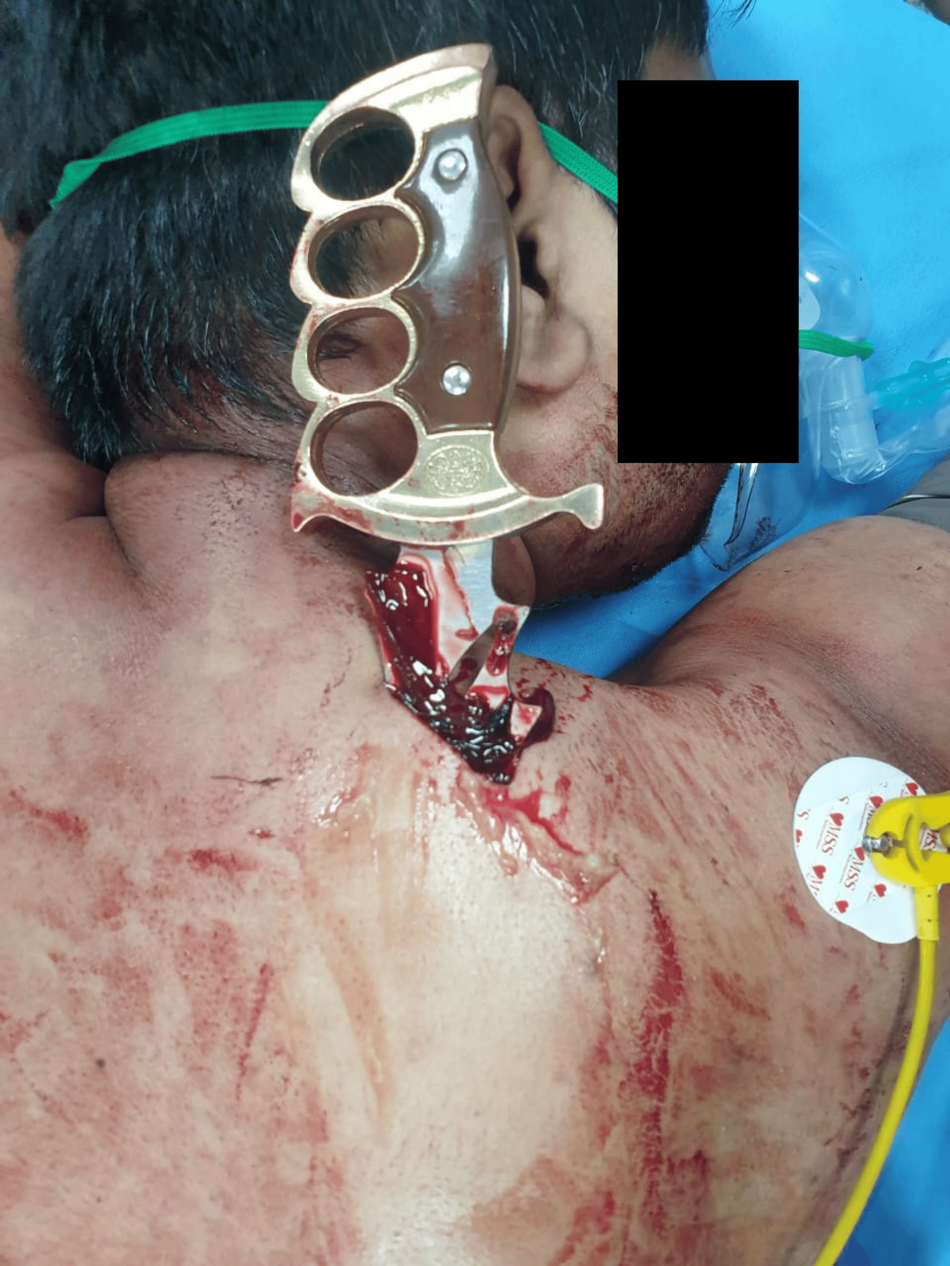
The presence of a knife lodged in the right scapular region.

A 32-year-old man was brought to the emergency room after an assault with a stab injury to the right scapular region (Figure 1).

**Figure 2:**
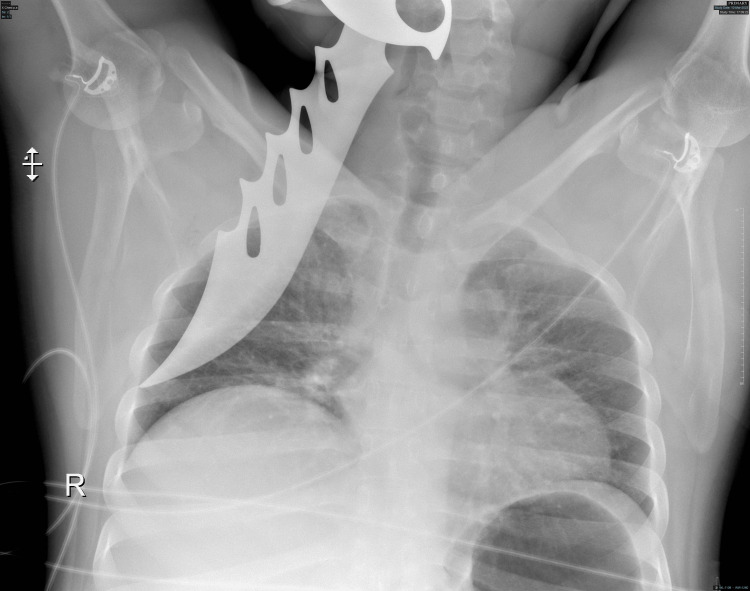
A chest X-ray showing an impaled knife on the right side of the chest.

He was found immobilized in the prone position with the knife still lodged in his back. Upon arrival, the patient had a Glasgow Coma Scale (GCS) score of 15 and was breathing comfortably on a 40% fraction of inspired oxygen (FiO_2_). He showed no signs of respiratory distress but was tachycardic and normotensive. Neurological examination was normal. The dorsal region revealed a knife embedded in the right scapular area with a 4 cm entry wound. The patient received 3 liters of crystalloid solution and 1 gram of tranexamic acid in the emergency room. A chest X-ray confirmed the presence of the impaled object on the right side without any additional injuries (Figure 2).

**Figure 3:**
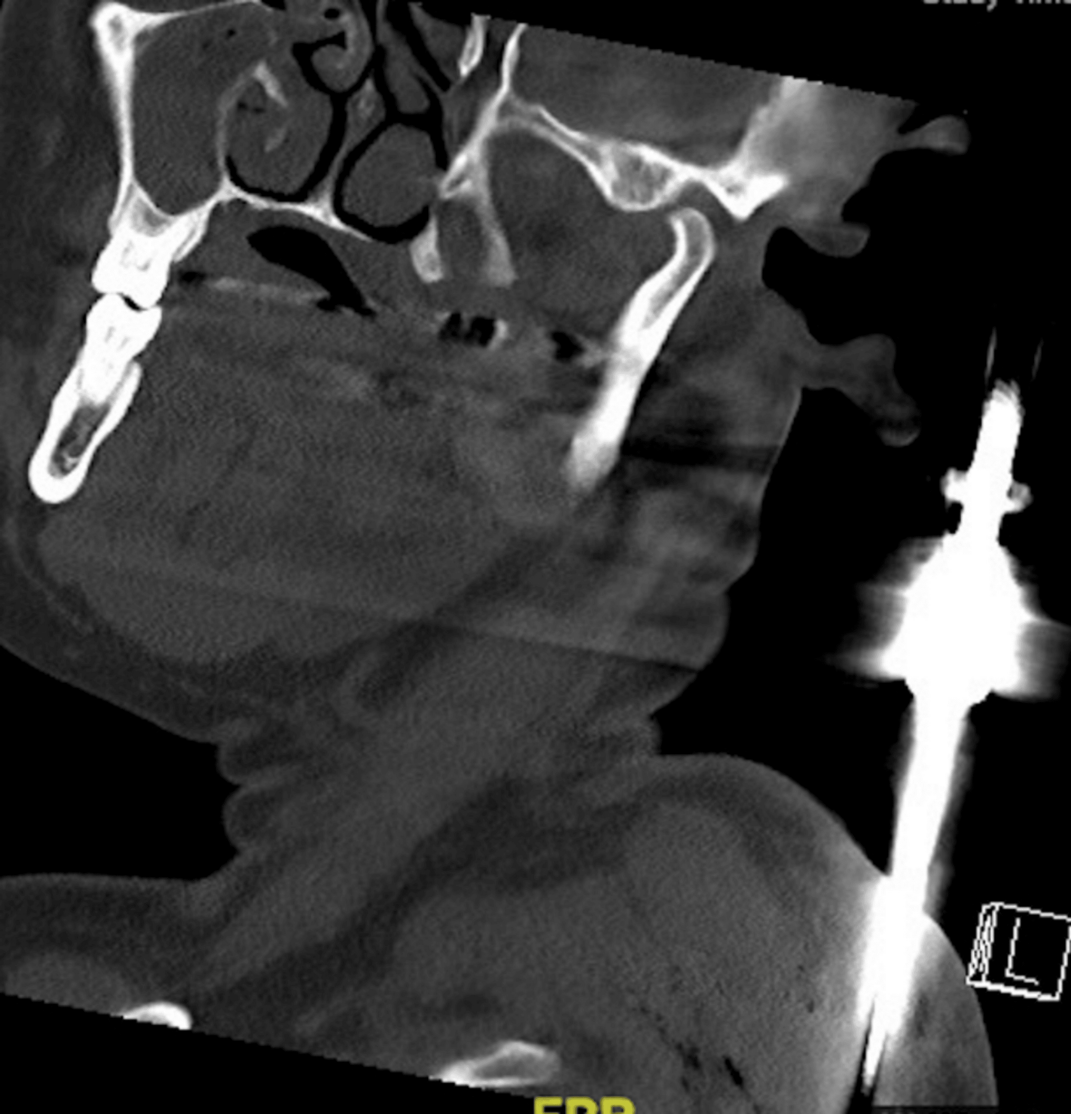
A CT scan of the thorax showing the knife lying along the medial border of the scapula.

A CT scan of the thorax revealed the knife positioned along the medial border of the scapula within the intermuscular plane (Figure 3).

Due to the risk of the knife shifting and exacerbating injuries, patient transfers were executed with precise coordination, utilizing sheets for support.

After a multidisciplinary evaluation, surgical removal of the knife under general anesthesia was planned. Considering the potential life-threatening risks associated with knife dislodgement, traditional supine and lateral decubitus positions were avoided for airway management. Alternative airway management plans were devised. The initial plan involved intubation in the prone position using a C-MAC® video laryngoscope (Karl Storz, Tuttlingen, Germany), with the head slightly rotated. Plan B involved using an I-gel® supraglottic airway device (Intersurgical Ltd., Wokingham, UK) for ventilation. Plan C proposed intubation in the lateral position, and plan D entailed performing a surgical airway if required.

**Figure 4:**
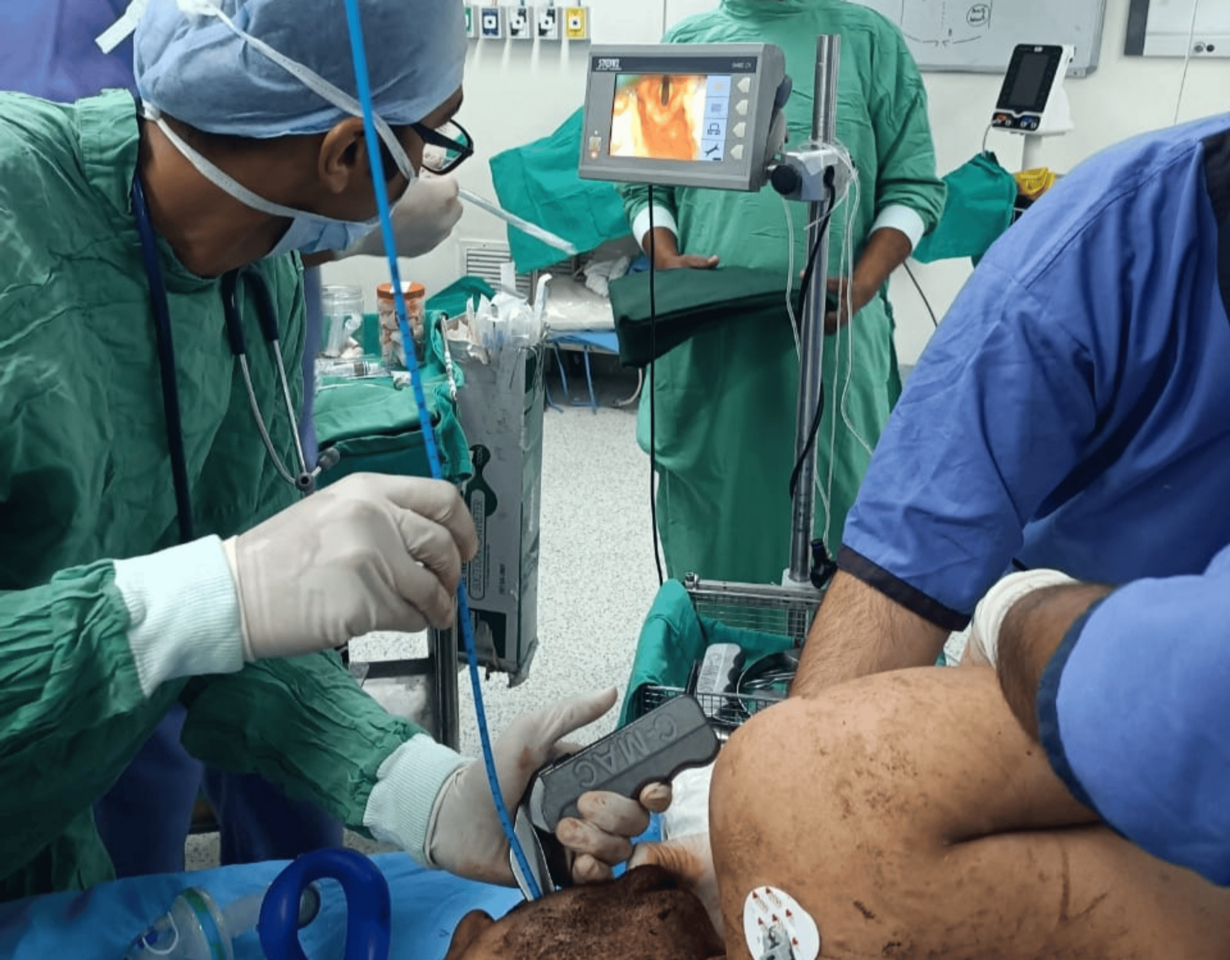
Bougie-guided intubation was performed with the assistance of a C-MAC® video laryngoscope.

The patient was monitored per the American Society of Anesthesiologists (ASA) standards and preoxygenated with 100% FiO_2_ for three minutes. Rapid sequence induction was performed with propofol (2 mg/kg) and succinylcholine (1.5 mg/kg). Ventilation with a single-person face mask and oropharyngeal airway was challenging. Using the C-MAC® video laryngoscope in the semi-prone position, with the head turned to the right, a Cormack-Lehane grade 2a glottis view was obtained. Bougie-guided intubation was successfully performed with an 8 mm cuffed orotracheal tube, confirmed via capnography (Figure 4).

The knife remained undisturbed throughout the procedure. The patient underwent successful surgical removal of the knife in the prone position. Following an uneventful intraoperative course, he was turned supine and extubated without complications.

Case 2

**Figure 5:**
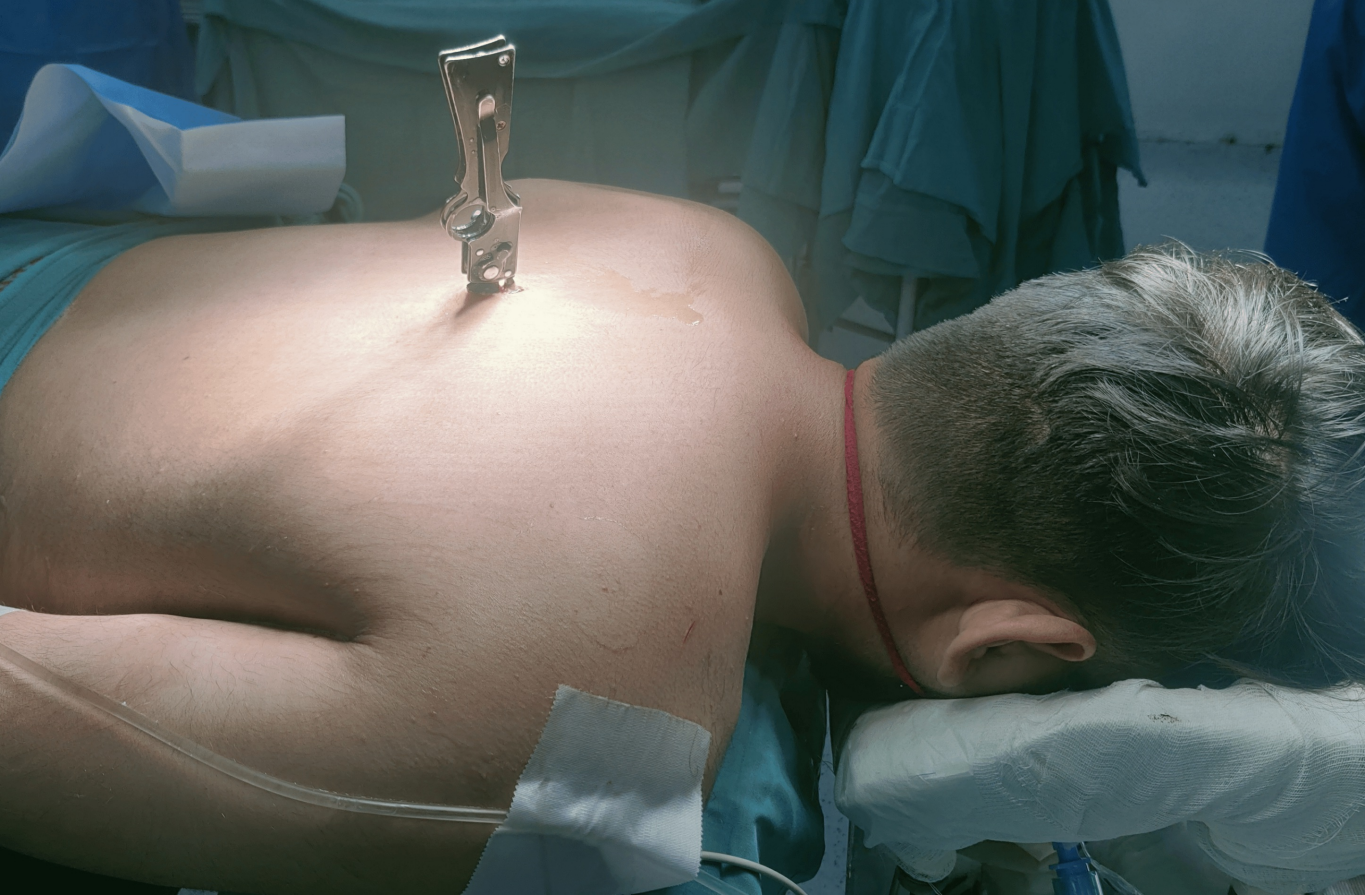
The presence of a knife lodged in the back of the patient.

A 30-year-old man, ASA physical status I, a victim of knife trauma, was brought to the emergency department in the prone position with a knife lodged in his back (Figure 5).

In the emergency room, the patient was alert and maintaining oxygenation on a face mask without clinical signs of respiratory distress. Hemodynamic parameters were normal. Neurologically, he exhibited monoplegia with no motor power in the left lower limb. Examination of the dorsal region revealed a knife deeply embedded in the center of the spine, below the shoulder blades. 

Due to the significant risk of the knife moving and exacerbating the injuries, a multidisciplinary team decided to surgically remove the knife. Considering the potential for life-threatening neurological complications, the use of supine and lateral decubitus positions for airway management was ruled out. Airway management plans were devised, with the primary approach involving the use of a C-MAC® video laryngoscope in the prone position with the head slightly rotated. A supraglottic airway device was kept as a backup, and a surgical airway was reserved as the last option.

**Figure 6:**
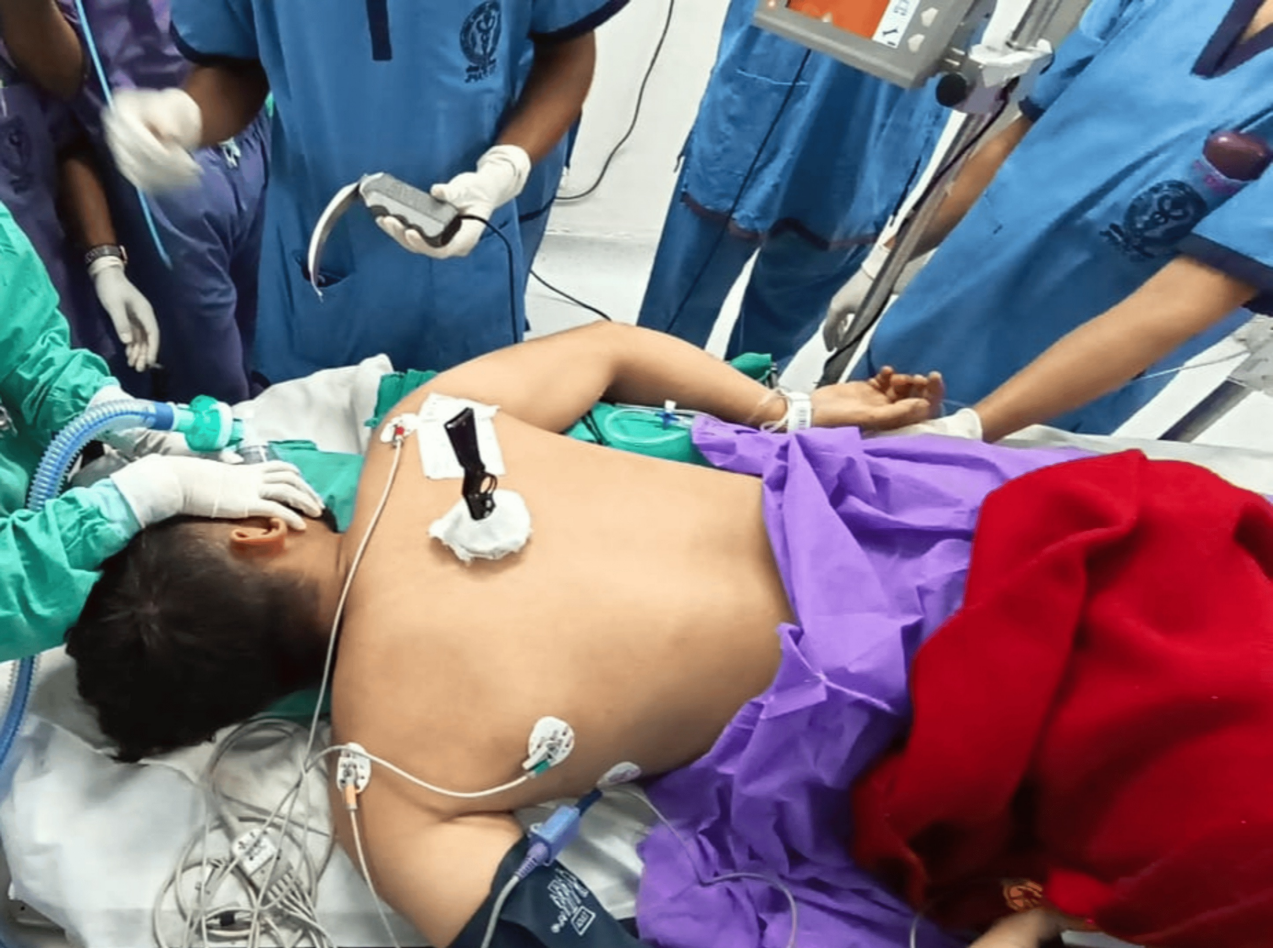
Bag-mask ventilation was performed in the prone position with the head turned to the right.

Transfers of the patient were executed with precision and coordination whenever necessary. ASA-standard monitors were applied, and the patient was preoxygenated with 100% oxygen for three minutes. Rapid sequence induction was performed with propofol (2 mg/kg) and rocuronium (1.2 mg/kg). Mask ventilation was challenging due to the prone position and the head being turned to the right (Figure 6).

**Figure 7:**
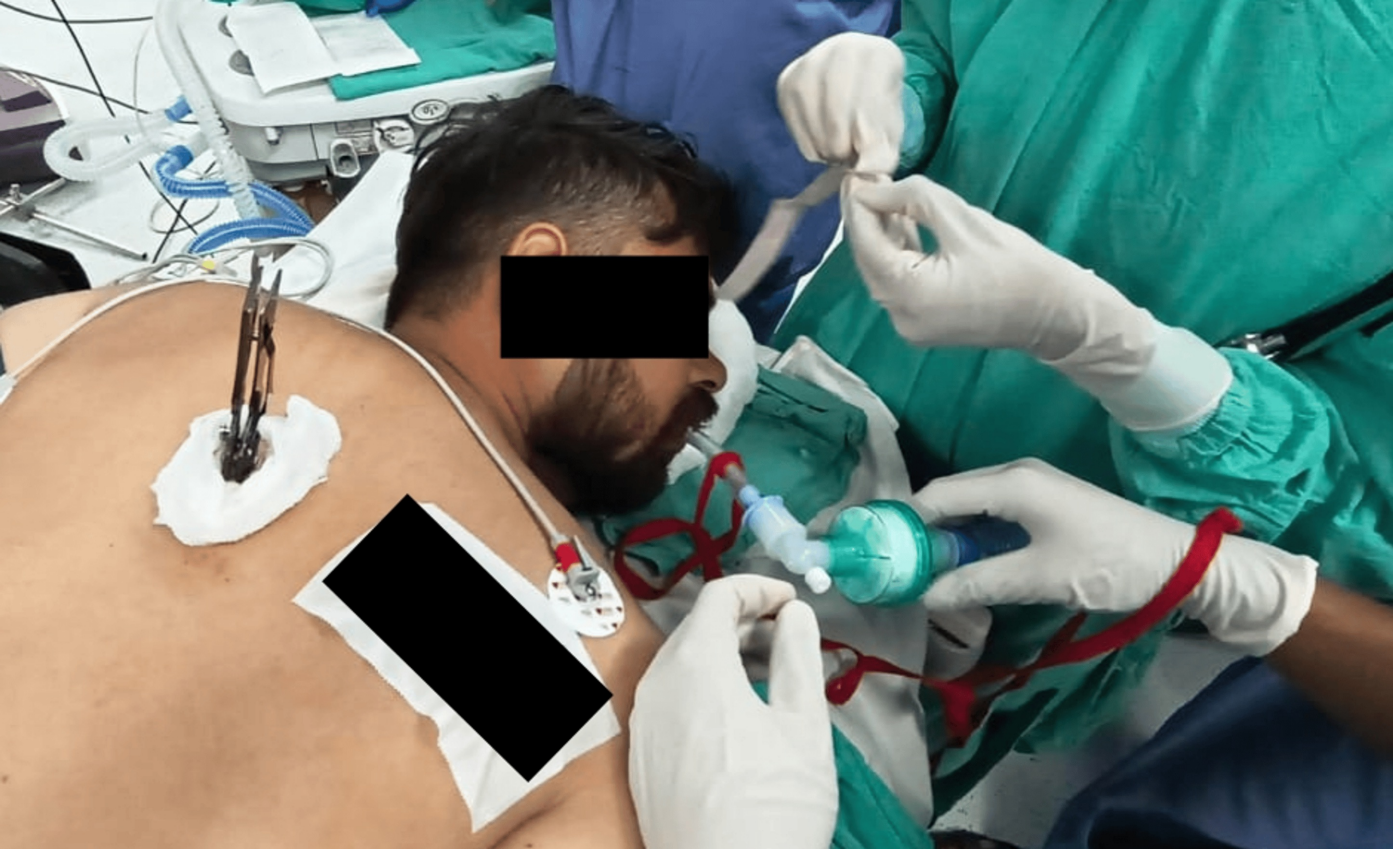
The airway was secured in the prone position.

The head was supported on sheets to achieve near-optimal positioning. Using the C-MAC® video laryngoscope in the prone position, the glottis view displayed on the screen was classified as Cormack-Lehane grade 2b. Intubation was successfully performed, and the airway was secured without dislodging the knife (Figure 7).

The patient underwent surgery for the careful removal of the knife. Following surgery, he was electively ventilated in the intensive care unit (ICU) and extubated successfully after two hours.

## Discussion

Stab wounds to the dorsal surface of the body have the potential to cause neurological harm. Although direct removal of the knife may be performed in patients with a normal neurological examination, surgical exploration is recommended if there is any suspicion or risk of spinal cord trauma [2]. Turning the patient to a supine or even lateral position in such injuries carries a risk of damaging vital structures such as the aorta, vena cava, cardiac chambers, lungs, and spinal cord.

When surgical exploration in the prone position is required, airway management becomes a significant challenge. Various approaches for managing the airway in the prone position have been described, including direct laryngoscopy with the head rotated, VL, fiberoptic intubation in awake patients, and the use of an LMA [3]. Baer and Nyström initially proposed routine intubation using a standard Macintosh laryngoscope in the prone position [4]. However, this technique has a steep learning curve and requires experienced anesthesiologists for effective execution. In contrast, VL in the prone position offers relative ease of use compared to direct laryngoscopy, as it provides a comprehensive visual display on a side screen. A Cochrane systematic review demonstrated that using the C-MAC® video laryngoscope, particularly in difficult airway situations, improves glottis visualization and reduces the number of failed intubations [5]. Additionally, the video laryngoscope with an extra-curved blade provides a superior view of the glottis.

Fiberoptic intubation in awake patients is considered one of the best options for managing difficult airways [6]. However, it requires extensive training, particularly for use in the prone position, and the time required to prepare the patient may limit its application in emergency trauma surgeries. Moreover, complications such as a bloody airway may limit its effectiveness due to poor visualization. The LMA has also been used successfully for airway rescue in prone patients. However, Abrishami et al. concluded that while there is sufficient evidence supporting the use of supraglottic airway devices in elective settings when the patient is prone, data is lacking for emergency situations [7]. It is also important to note that these devices do not guarantee protection against aspiration and the risk of dislodgement is higher in the prone position.

Although fiberoptic scopes and intubating LMAs have been documented for prone intubation, video laryngoscope-guided intubation is considered more practical once proficiency is achieved, due to its broader availability compared to fiberoptic devices. In the case discussed here, considering the prone position and the absence of other difficult airway indicators, we initially attempted orotracheal intubation using the C-MAC® video laryngoscope following rapid sequence induction. Backup airway management plans were also formulated. The use of VL for intubation in the prone position remains relatively underexplored. To date, only one case has been reported by Gaszynski, detailing the use of an Airtraq Avant video laryngoscope (Prodol Meditec S.A., Vizcaya, Spain) in the prone position [8]. However, we successfully intubated both patients with the help of a video laryngoscope. The results of this case report should be interpreted with caution, as intubation in an unusual position may compromise safety. Every effort should be made to perform awake fiberoptic intubation, depending on the clinical situation, expertise, and resource availability.

Endotracheal intubation in the prone position poses challenges due to restricted access to the patient's head and limited space around the mouth. As a result, non-channeled video laryngoscopes are preferred, as they occupy less space and are easier to insert compared to channeled devices. In the prone position, the video laryngoscope is maneuvered differently compared to the supine position; the blade is pulled downward to elevate the epiglottis. This technique may confuse inexperienced operators. Therefore, the operator should be well-trained and proficient in the use of the chosen video laryngoscope [9]. The ultimate decision on the method of airway management should depend on the resources available in the operating theatre and the anesthetist's expertise and preference in such situations.

## Conclusions

Stab wounds to the dorsal surface can pose a risk of neurological harm, especially if spinal cord trauma is suspected. Surgical exploration in the prone position is often necessary, but this presents significant airway management challenges. Various methods, including direct laryngoscopy, VL, fiberoptic intubation, and LMA, have been described for airway management in the prone position. VL is a practical option due to its superior glottis visualization and ease of use compared to direct laryngoscopy or fiberoptic intubation, which requires extensive training and patient cooperation. This case highlights the successful use of the C-MAC® video laryngoscope for prone intubation. However, the ultimate decision on the method of airway management should depend on the resources available in the operating theatre, as well as the anesthetist's expertise and preference in such situations. Every effort should be made to perform awake intubation whenever possible.
